# Machine learning molecular dynamics reveals the structural origin of the first sharp diffraction peak in high-density silica glasses

**DOI:** 10.1038/s41598-023-44732-0

**Published:** 2023-11-16

**Authors:** Keita Kobayashi, Masahiko Okumura, Hiroki Nakamura, Mitsuhiro Itakura, Masahiko Machida, Shingo Urata, Kentaro Suzuya

**Affiliations:** 1 CCSE, Japan Atomic Energy Agency, Kashiwa, Chiba 277-0871 Japan; 2 Innovative Technology Research Center, AGC Inc., 1150 Hazawa-cho, Kanagawa-ku, Yokohama, Kanagawa 221-8755 Japan; 3 Materials and Life Science Division, J-PARC Center, Japan Atomic Energy Agency, Tokai, Ibaraki 319-1195 Japan

**Keywords:** Chemical physics, Molecular dynamics, Computational chemistry, Theory and computation

## Abstract

The first sharp diffraction peak (FSDP) in the total structure factor has long been regarded as a characteristic feature of medium-range order (MRO) in amorphous materials with a polyhedron network, and its underlying structural origin is a subject of ongoing debate. In this study, we utilized machine learning molecular dynamics (MLMD) simulations to explore the origin of FSDP in two typical high-density silica glasses: silica glass under pressure and permanently densified glass. Our MLMD simulations accurately reproduce the structural properties of high-density silica glasses observed in experiments, including changes in the FSDP intensity depending on the compression temperature. By analyzing the simulated silica glass structures, we uncover the structural origin responsible for the changes in the MRO at high density in terms of the periodicity between the ring centers and the shape of the rings. The reduction or enhancement of MRO in the high-density silica glasses can be attributed to how the rings deform under compression.

## Introduction

Silicon dioxide is an essential material used not only in glass manufacturing but also in the production of semiconductors^1,2^. In addition to its industrial significance, silica glass is of great interest in fundamental research as an archetypal polyhedron network former^3^. One of the most fundamental issues of the polyhedron network forming materials is the origin of the first sharp diffraction peak (FSDP) in the total structure factor measured by neutron and X-ray diffraction (ND and XRD) experiments. The FSDP has been considered a signature of medium-range order (MRO) in disordered materials. Numerous models have been proposed to explain the relationship between the FSDP and the underlying atomic structures^4^. The periodicity of the quasi-crystalline^5,6^, layered structures^7,8^, cluster-like regions^9,10^, and chemical ordering of the voids around cations^11,12^ have been explored as the structural origins of FSDP. The MRO and FSDP in amorphous materials have also been discussed in relation to their glass-forming ability^13–16^. In the case of silica glass, the quasi-periodicity formed in the SiO$_{4}$ tetrahedral network is considered to induce FSDP in the total structure factor^17–26^.

The FSDP is significantly affected by the density of silica glass^24,27–31^. For example, it is well known that the FSDP heights of the permanently densified silica glass produced by cold compression^27,28^ and the silica glass under high pressure^29,31^ are significantly reduced, suggesting that the densification disrupts the MRO in the glasses. By contrast, Onodera et al.^24^ recently reported unusual behavior of FSDP in high-density silica glass. They have shown that the permanently densified silica glass created via hot compression enhances the FSDP. Depending on the thermal treatment used for the formation of high-density silica glass, the FSDP exhibits the opposite behavior, which cannot be determined solely by the density. Thus, investigating high-density silica glass is crucial for understanding the origin of FSDP in disordered polyhedral network materials.

Reliable information in real space is essential for detailed investigations of the structural properties of disordered materials. However, XRD and ND experiments on noncrystalline materials can only provide one-dimensional structural information, such as total structure factors and their corresponding pair distribution functions. Therefore, computational methods, including the reverse Monte Carlo method, molecular dynamics (MD) simulations, and first-principles calculations based on the density functional theory (DFT), have been extensively used to obtain three-dimensional real-space structural information on disordered materials^20,23,24,32–38^.

Over the past decade, a variety of machine learning techniques have been applied to predict material properties and characterize the structures of glasses^39–44^. Specifically, machine learning molecular dynamics (MLMD) is a breakthrough in the accurate structural prediction of materials^45–53^. In MLMD, flexible functions with several adjustable parameters are employed as interatomic potentials for MD simulations: for example, artificial neural networks^45,46^ and Gaussian processes^47,48^. These are referred to as machine-learning potentials. Machine-learning potentials are trained with numerous DFT calculations to imitate the DFT potential-energy surface and enable large-scale MD simulations with near DFT accuracy. Machine-learning potentials for covalent glasses and liquids have been constructed using linear regression^49^, artificial neural networks^49–52^, and the Gaussian approximation potential^53^. In particular, Erhard et al.^53^ revealed that a machine-learning potential based on DFT with the strongly constrained and appropriately normed meta-GGA exchange-correlation functional^54^ enabled high-accuracy calculations for various phases of silica. MLMD simulations, which can provide accurate real-space structural information with almost DFT accuracy, are expected to further our understanding of MRO in disordered materials.

In this study, we investigated the detailed microscopic structures as the origin of MRO in high-density silica glass using MLMD simulations with neural networks type machine-learning potential^45,46^trained using the results of DFT calculations with the strongly constrained and appropriately normed meta-GGA exchange-correlation functional. We aimed to elucidate the relationship between the FSDP and the atomic structures of high-density silica glass. To achieve this outcome, we subjected silica glass to different types of deformations, as performed in previous experiments^24,27,29,31^. We primarily focused on two types of high-density silica glass: silica glass under pressure (SGUP) at room temperature and densified silica glass created by hot compression (DSG). Elastic deformation is expected to dominate the deformation of the SGUP, whereas plastic deformation with Si–O bond recombination is expected to predominantly govern DSG formation. Investigating the difference in the deformation behavior between SGUP and DSG is expected to deepen our understanding of MRO in silica glass. We show that the MLMD simulations reasonably reproduce various experimental results for high-density silica glasses, including the enhancement (or reduction) of FSDP. The structural origin of MRO in the simulated high-density silica glasses was investigated from the perspective of the quasi-periodicity of the boundaries between successive cages formed by rings in the SiO$_4$ tetrahedral network. The difference between the structural origins of the MROs in the SGUP and DSG was clarified by examining the deformation manner of the rings under cold and hot compression.

## Results

**Table 1:** Structural properties of the OSG computed by MLMD.

	MLMD	Exp.
Density [g/cm${}^{2}$]	2.245	2.196^55^
$d_{\textrm SiO}$ [Å]	1.61	1.61^19^
$d_{\textrm SiSi}$ [Å]	3.08	3.07^19^
$d_{\textrm OO}$ [Å]	2.62	2.62^19^
$\theta _{\textrm OSiO}$ [$^{\circ }$]	109.41	109.47^56^
$\theta _{\textrm SiOSi}$ [$^{\circ }$]	144.57	148.3^56^, 146^32^

The Si–O, Si–Si, and O–O bond lengths ($d_{\textrm SiO}$, $d_{\textrm SiSi}$, and $d_{\textrm OO}$) are determined as the first-peak positions of pair-distribution functions. The averaged O–Si–O and Si–O–Si angles ($\theta _{\textrm OSiO}$ and $\theta _{\textrm SiOSi}$) are defined using the mean value of the angle distributions.

**Figure 1:**
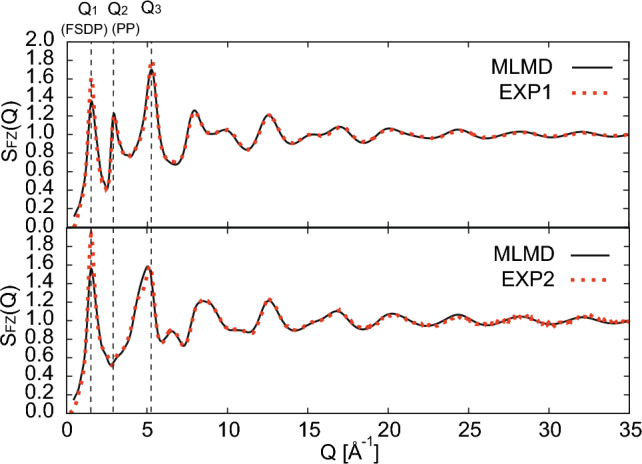
Faber–Ziman structure total factor $S_{\textrm FZ}(Q)$ of the OSG obtained via the MLMD melt-quenching simulation and experiment (Exp1^24^ and Exp2^57^). The upper and lower panels show the results of the ND and XRD, respectively.

The structural properties of the ordinary silica glass (OSG) obtained via MLMD melt-quenching simulations are summarized in Table 1. The percentage error of the density in the experimental data was approximately 2.4 %, and the obtained bond lengths agreed well with the experimental results. The O–Si–O angle is extremely close to the central angle of the ideal tetrahedron, 109.47$^{\circ }$. The Si–O–Si angle obtained through MLMD was comparable to the experimental data. Figure 1 shows the Faber–Ziman total structure factor $S_{\textrm FZ}(Q)$ of the simulated and experimental OSGs. Three distinctive peaks, Q1, Q2, and Q3, were observed for $S_{\textrm FZ}(Q)$. The first peak $Q_1$ is the FSDP, reflecting the MRO embedded in the SiO$_4$ tetrahedral networks. The second peak $Q_{2}$ is called as the principal peak (PP). The scale of the $Q_2$ agrees with the heights of the SiO$_4$ tetrahedra as $Q_2 \simeq 2\pi /(4d_{\textrm SiO}/3)$, and the PP is considered to reflect the local arrangement of the SiO$_4$ tetrahedra^58,59^. The third peak $Q_3$ is a generic feature of amorphous materials arising from single-pairwise correlations between nearest-neighbor atoms^23,59^. Although the total structure factor computed using MLMD slightly underestimates the height of the FSDP, all the peak positions of the $S_{\textrm FZ}(Q)$ show excellent agreement with the experimental data from low to high scattering vector *Q*. The structures of the simulated silica glass generated by MLMD simulations with near DFT accuracy were considered reliable for discussing the structural properties, including MRO.

**Figure 2:**
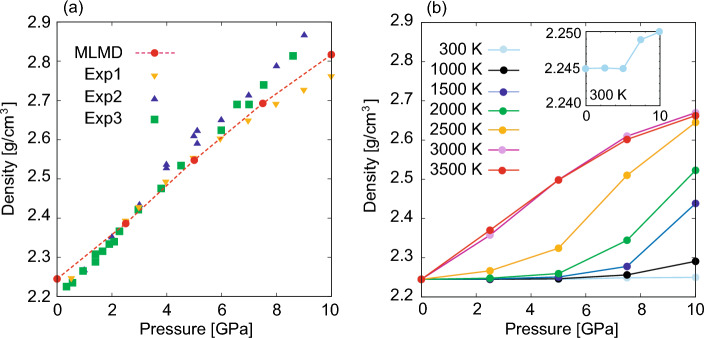
(**a**) Density change of the SGUP at 300 K. Circle dots denote the results obtained via MLMD. Square (Exp1), triangle (Exp2), and inverted triangle dots (Exp3) represent the experimental data^60–62^. (**b**) Density of the DSGs compressed at different pressures and temperatures.

Based on MLMD simulations, we analyzed the structural properties of two typical high-density silica glasses: SGUP and DSG. The pressure-density curve of the SGUP evaluated via MLMD was consistent with the trend of the experimental data, as shown in Fig. 2a. Figure 2b shows the density of the DSG created by compressing the OSG at various pressures and temperatures. Although the plastic deformation of silica glass at room temperature was experimentally observed at approximately 9 GPa^60,61^, the density of the silica glass compressed at 10 GPa and 300 K was extremely low in the MLMD simulation (see the inset of Fig. 2b). This was due to the limitation of the time scale of the MD simulation, and compression at high temperatures was required to achieve a large structural relaxation of silica glass^38,63–65^. On the timescale of the present simulation, densification due to hot compression above 1000 K can be detected. Sufficient structural relaxation of silica glass is considered to be realized above 3000 K, because the density of silica glass does not show a temperature dependence above 3000 K. Experimentally, silica glass can be densified up to approximately 23%, the value of which depends on the compression pressure and temperature^24,28,30,66,67^. In the present simulation, with hot compression up to 10 GPa and 3500 K, the silica glass was densified by up to 19%.

**Figure 3:**
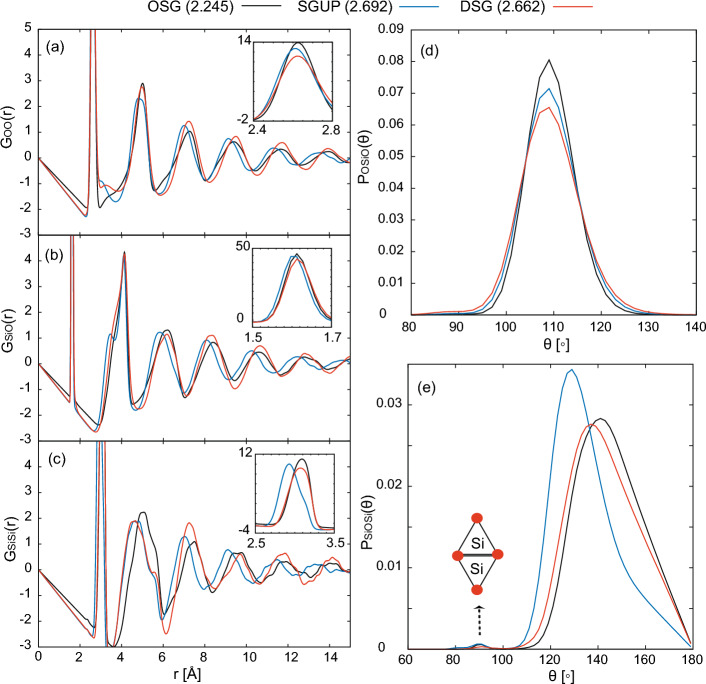
(**a**), (**b**), and (**c**): Partial differential distribution functions $G_{\alpha \beta }(r)$ for O–O, Si–Si, and Si–O pair. The insets show the first peak of $G_{\alpha \beta }(r)$. (**d**) and (**e**): O–Si–O and Si–O–Si angle distributions. The inset in (**e**) denotes the edge-sharing tetrahedron that contributes to the Si–O–Si angle distribution at approximately 90$^\circ$. The distributions of OSG, SGUP, and DSG are indicated by the black, blue, and red lines, respectively. The values in $(\cdot )$ in the legends represent the density of the silica glass [g/cm$^{3}$].

**Figure 4:**
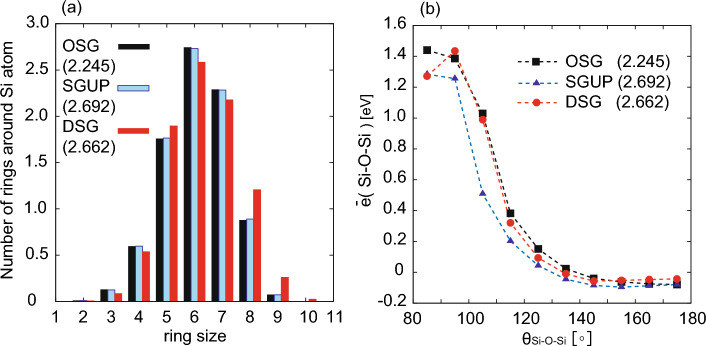
(**a**) Ring-size distribution around silicon atom for OSG, SGUP, and DSG. (**b**) Mean Si–O–Si atomic energy, $\bar{e}(\text {Si--O--Si})$ vs Si–O–Si angle, $\theta _{\text {Si--O--Si}}$. The Si–O–Si atomic energy is calculated from machine-learning potential atomic energy *e*(*i*) as $\bar{e}(\text {Si--O--Si}) = \sum _{i\in \{\text {Si--O--Si}\}} (e(i)-\bar{e}(i))$, where $\bar{e}(i)$ is mean atomic energy of silicon and oxygen atoms. The values in $(\cdot )$ in the legends denote the density of silica glass [g/cm$^{3}$].

Figure 3 shows the partial differential distribution $G_{\alpha \beta }(r)$ and angle distribution functions $P_{\alpha \beta \gamma }(\theta )$ of the OSG, the SGUP, and the DSG. The partial differential distribution function^19,33^ is defined as $G_{\alpha \beta }(r) = 4\pi \rho _{0}r[g_{\alpha \beta }(r)-1]$, where $\rho _{0}$ is the density of the silica glass. Herein, we focus on the SGUP and the DSG with almost the same density (2.692 and 2.662 [g/cm$^{3}$], respectively). The first peak positions of $G_{\textrm OO}(r)$ and $G_{\textrm SiO}(r)$ in SGUP and DSG, which correspond to the O–O and Si–O bond lengths, were almost unchanged from those of OSG. All the peaks of the O–Si–O angle distribution functions are close to 109$^\circ$, as shown in Fig. 3b. These results indicated that the structures of the SiO$_{4}$ tetrahedral units were well maintained under cold and hot compression. The peak of the Si–O–Si angle distribution of the SGUP largely shifted toward a lower angle than that of the OSG, whereas the corresponding peak shift of the DSG was small. In all cases, the contribution of the edge-sharing SiO$_4$ tetrahedra was negligible (see inset of Fig. 3e), and the SiO$_4$ networks of the glasses consisted of corner-sharing tetrahedral SiO$_{4}$. Overall, the peaks of $G_{\alpha \beta }(r)$ of the SGUP shifted to a lower position from those of the OSG, except the first peak. In addition, the ring size distribution of the SGUP was almost unchanged from that of the OSG, as shown in Fig. 4a. The structure of the SGUP is considered to have almost the same topology as that of the OSG with a reduced Si–O–Si angle. By contrast, in the case of the DSG, there is no regular trend with regard to the peak shifts of $G_{\alpha \beta }(r)$ from those of the OSG. Furthermore, the ratio of rings larger than seven-membered rings increased in the DSG compared to that in the OSG (see Fig. 4a). These results indicate that the structure of DSG has a different topology from those of OSG and SGUP because of the recombination of Si–O bonds by compression at high temperatures. The increase in the number of larger rings agrees with the reverse Monte Carlo result for the DSG created by hot compression^24^. Notably, the creation of larger rings in the DSG is linked to a small change in the Si–O–Si angle distribution of the DSG (Fig. 3e). This is because large rings can adopt various configurations to allow large Si–O–Si angles, whereas the configurations of small rings are more constrained, and the deformation of small rings inevitably leads to a reduction in the Si–O–Si angle. From an energy perspective, a small Si–O–Si angle increases the local energy related to the Si–O–Si bond, as shown in Fig. 4b. Therefore, large rings were generated in the DSG to avoid the local energy increment owing to the significant reduction in the Si–O–Si angle caused by compression.

**Figure 5:**
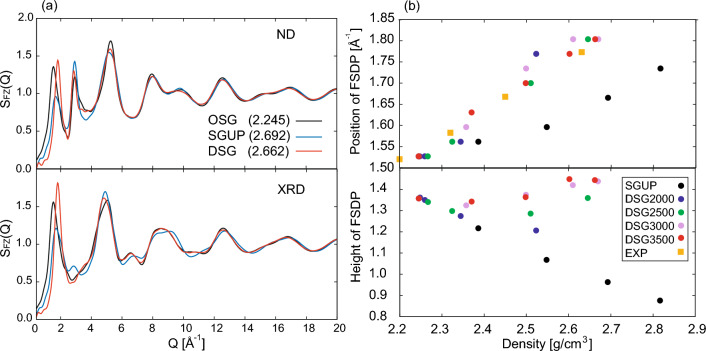
(**a**) Faber–Ziman total structure factor $S_{\textrm FZ}(Q)$ for ND and XRD computed via MLMD simulation. The black, blue, and red lines represent the structure factors for OSG, SGUP, and DSG, respectively. The values in $(\cdot )$ in the legends denote the density of silica glass [g/cm$^{3}$]. (**b**) shows the density dependence of position (upper panel) and height (lower panel) of the FSDP in the Faber–Ziman structure factor of ND. The black dots denote the results of the SGUP. The blue, green, purple, and red dots represent the results of the DSGs compressed at 2000, 2500, 3000, and 3500 K (DSG2000, DSG2500, DSG3000, and DSG3500), respectively. The orange squares denote the experimental data of the DSG^27^.

Figure 5a shows the Faber–Ziman total structure factors $S_{\textrm FZ}(Q)$ for SGUP and DSG with approximately the same density. The FSDP intensities of SGUP and DSG showed reduction and enhancement, respectively, which are in good agreement with previous experimental reports^24,29,31^ (see also Supplementary Information). In particular, MLMD successfully reproduced the enhancement of the FSDP in DSG, which was recently reported by Onodera et al.^24^. The density dependence of the positions and heights of the FSDPs in the SGUP and DSGs compressed at various temperatures is shown in Fig. 5b. The positions of the FSDPs shifted towards a higher wavevector with increasing density in all cases, and the shifts of the DSGs were larger than those of the SGUP at almost the same density. Although the positions of the FSDP in the DSGs were slightly scattered, they were assumed to be proportional to the density, as observed in the experimental results^27^. Regarding the density dependence of the height of the FSDP, although the height of the FSDP in the SGUP monotonically decreased with increasing density, those in the DSGs showed a reduction or enhancement depending on the compression temperature. As reported in the experiments, FSDP in DSG was sensitive to thermal treatment^24^. The PPs in the $S_{\textrm FZ}(Q)$ observed by the ND became sharper with increasing density, which was considered to be related to the oxygen packing fraction^23,58,59^. Although the PP usually does not appear in the XRD structure factor as in the case of the OSG and DSG, the $S_{\textrm FZ}(Q)$ observed via XRD for the SGUP reveals a small peak around 2.9 Å${}^{-1}$, which is also observed in in-situ high-pressure experiments^31^.

## Discussion

**Figure 6:**
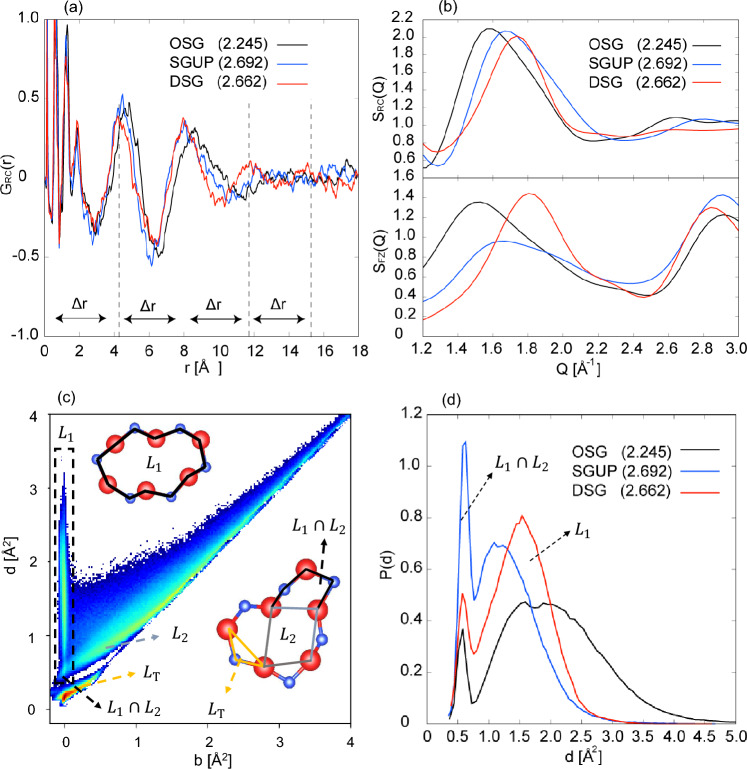
(**a**) Ring center differential distribution function $G_{\textrm RC}(r)$. (**b**) Upper panel shows the ring center structure factor and the lower panel represents the Faber–Ziman total structure factor of ND. (**c**) One-dimensional persistence diagram of the DSG with density 2.662 [g/cm$^{3}$]. The horizontal and vertical axes (birth and death) represent the length scale of loops embedded in atomic configuration. (**d**) Probability distribution of death scale on the $L_{1}$ band (region enclosed by the black dashed line in (**c**)) The values in $(\cdot )$ in the legends denote the density of silica glass [g/cm$^{3}$].

We have demonstrated that the MLMD simulations accurately reproduced the experimental results for high-density silica glasses. Based on reliably simulated high-density silica glasses, we discuss the structural origin of the FSDP. The FSDP in silica glass has been considered to arise from the quasi-periodicity of the boundaries between successive cages formed by the rings in the SiO$_4$ tetrahedral network^17–20^. To extract the quasi-periodicity of the boundaries from the simulated glasses, we evaluated the ring center pair distribution function defined as

1 $$\begin{aligned} g_{\textrm RC}(r) = \sum _{i,j(\ne i)} \frac{V\delta (r- |\varvec{r}_{{\textrm RC},j}-\varvec{r}_{{\textrm RC},i}|)}{4\pi r^2 N_{\textrm RC}(N_{\textrm RC}-1)}, \end{aligned}$$

where *V* is the system volume, $N_{\textrm RC}$ denotes the total number of the rings obtained by shortest path analysis, and $\varvec{r}_{{\textrm RC},i}$ indicates the centroid coordinate of *i*-th ring. Figure 6a shows the ring center differential distribution function, $G_{\textrm RC}(r)=4\pi \rho _{\textrm R} r[g_{\textrm RC}(r)-1]$, where $\rho _{\textrm RC} = N_{\textrm RC}/V$ The peaks of $G_{\textrm RC}(r)$ in the short-range region within 3Å are attributed to the overlapping of some centroid positions of the rings. The distinctive peaks appear in the intermediate range (3Å$\le r$) with a period $\Delta r \simeq 4$, which coincides with the scale of the FSDP, $Q_{1}\simeq 2\pi /\Delta r$. We evaluated the ring center structure factor $S_{\textrm RC}(Q)$, defined by the Fourier transform of $g_{\textrm RC}(r)$, and the results are shown in Fig. 6b. The peak positions of $S_{\textrm RC}(Q)$ qualitatively reproduced the positions of the FSDPs in the total structure factor $S_{\textrm FZ}(Q)$. This result supports the conclusion of the previous studies that the origin of the FSDP is the quasi-periodicity between the succession of cages^17–20^. Although the ring center structure factor $S_{\textrm RC}(Q)$ does not reproduce the change in the height of the FSDP in $S_{\textrm FZ}(Q)$ , it can capture some characteristic features of the MRO in high-density silica glasses. The shapes of $S_{\textrm RC}(Q)$ for the OSG and SGUP are almost identical, and $S_{\textrm RC}(Q)$ for the SGUP is regarded as a simple shift of the OSG to a higher scattering vector, which is consistent with the trend of the partial differential distribution functions of the SGUP (see Fig. 3a–c). This result implies that the MRO in the SGUP and OSG are basically identical. By contrast, the peak of $S_{\textrm RC}(Q)$ of DSG is sharper than that of OSG and SGUP. The sharper peak indicates a clearer quasi-periodicity between the cage boundaries, which can also be confirmed by the long-lasting periodicity of $G_{\textrm RC}(r)$ of the DSG, as shown in Fig. 6a.

The ring center structure factor $S_{\textrm RC}(Q)$ does not reproduce the changes in the height of the FSDPs with increasing density because it only considers the correlation between ring centers and ignores information on the shapes of the rings. To address this issue, we used a persistence diagram to analyze the shapes of the rings in high-density silica glasses. Figure 6c shows the one-dimensional persistence diagram of the DSG with three characteristic bands: $L_{\textrm T}$, $L_1$, and $L_2$. The $L_{\textrm T}$ band represents a triangular loop comprising silicon and its two nearest oxygen atoms, indicating a short-range order owing to the local structure of the SiO$_{4}$ tetrahedron (right lower panel in Fig. 6c). In the persistence diagram calculations, we defined the initial radii of the atomic balls for silicon $r_{\textrm Si}$ and oxygen $r_{\textrm O}$ to satisfy the condition $d_{\textrm SiO} = r_{\textrm Si} + r_{\textrm O}$. Therefore, the rings obtained through shortest path analysis based on Si–O bond length appear nearly on the vertical line along $b \simeq 0$ in the $L_{1}$ band (the left upper subpanel in Fig. 6c). The $L_{2}$ band represents the subloops arising within the loops in the $L_{1}$ band and continuing into the $L_{1}$ band. We refer to the intersecting region of the $L_{1}$ and $L_{2}$ bands as $L_{1}\cap L_{2}$ band, which includes loops corresponding to largely distorted ring structures, as shown in the right lower panel of Fig. 6c. Figure 6d shows the probability distribution of the death scale for the $L_{1}$ band. Both the $L_{1}$ peaks of SGUP and DSG shift towards a smaller death scale, indicating that the mean ring radii are reduced in the high-density silica glasses. The primary difference between the SGUP and the DSG indicates the peak development related to the $L_{1}\cap L_{2}$ band ($d\simeq 0.5$). In the SGUP, the peak of the $L_{1}\cap L_{2}$ band significantly develops, and the boundary between the $L_{1}$ and the $L_{1}\cap L_{2}$ bands is rendered ambiguous. This result suggests that compression causes extensive distortion of the ring shapes in the SGUP, which is consistent with the considerable reduction in the Si–O–Si angles illustrated in Fig. 3e. Consequently, the boundary surfaces of the cages formed by the rings in the SGUP are significantly disturbed compared to those in the OSG and DSG, which is considered to weaken the quasi-Bragg diffraction between the boundaries and decrease the FSDP. By contrast, the $L_{1}\cap L_{2}$ peak of the DSG is small, and the $L_{1}$ peak becomes shaper. This indicates that structural relaxation accompanied by hot compression suppresses the creation of distorted rings caused by densification, resulting in more aligned boundary surfaces of successive cages in the DSG. The cage boundary surfaces of the DSG, which consist of rings with well-aligned length scales and small disturbances, enhance the quasi-Bragg diffraction related to the FSDP.

**Figure 7:**
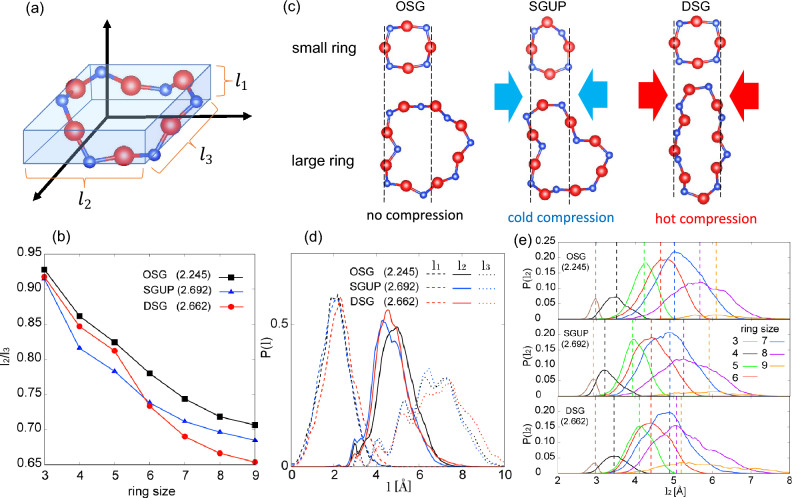
(**a**) Ring approximated as a cuboid on the principal axes of the moment tensor of a ring. $l_1$, $l_2$, and $l_3$ represent the length of the sides of the cuboid ($l_1\le l_2 \le l_3$). (**b**) Averaged ratio of the sides of the cuboid $l_{2}/l_{3}$ for each ring size. (**c**) Schematic figures of ring deformation for small and large rings of SGUP and DSG. (**d**) Distribution of $l_1$, $l_2$, and $l_3$ for OSG, SGUP and DSG. (**e**) Distribution of $l_2$ calculated for each ring size. Dashed lines represent the peak position of the $l_2$ distribution for guide. The values in $(\cdot )$ in the legends denote the density of silica glass [g/cm$^{3}$].

Finally, we uncover the underlying mechanism for developing the MRO, focusing on ring deformation caused by compression. We characterized deformation of the ring shape using a cuboid approximation of a ring on the ring pseudo-plane. To define the pseudo-plane for each ring, we computed the inertia tensor of the *i*-th ring as

2 $$\begin{aligned} M^{(i)} = \sum _{j\in i\text {-th ring}} \begin{pmatrix} y_{j}^{2} + z_{j}^{2} &{} -x_{j} y_{j} &{} -x_{j} z_{j} \\ -x_{j} y_{j} &{} x_{j}^{2} + z_{j}^{2} &{} -y_{j} z_{j} \\ -x_{j} z_{j} &{} -y_{j} z_{j} &{} x_{j}^{2} + y_{j}^{2} \\ \end{pmatrix} \end{aligned}$$

We then determined the axes of each ring’s pseudo-plane using the principal axes of its inertia tensor. The lengths of the sides of the *i*-th ring approximated as a cuboid were defined as follows:

3 $$\begin{aligned} l_{k}^{(i)} = \max (\{ [U^{(i)}\varvec{r}_{j\in i\text {-th ring}}]_k \}) - \min (\{ [U^{(i)}\varvec{r}_{j\in i\text {-th ring}}]_k \}), \end{aligned}$$

where $U^{(i)}$ is the matrix to diagonalize $M^{(i)}$ and the index *k* denotes the principal axes of the inertia tensor ($k=1,2,3$). The ordering of $l_{k}^{(i)}$ is such that $l_{1}^{(i)}\le l_{2}^{(i)} \le l_{3}^{(i)}$, where $l_1^{(i)}$ represents the thickness of the pseudo-plane of the *i*-th ring, $l_2$ and $l_3$ characterize the shape of the ring within the pseudo-plane (see Fig. 7a), respectively. To quantify the deformation of the ring shape, we evaluated the average aspect ratio of the sides, $l_2/l_3$, of the cuboids approximating the ring shapes. The aspect ratio, $l_2/l_3$, indicates the shape of a ring in the pseudo-plane, where a value close to 1 suggests that the shape of the ring is close to isotropic in the pseudo-plane, whereas a small value of $l_2/l_3$ indicates that the ring has an elongated shape. The aspect ratio, $l_2/l_3$, revealed noteworthy differences between the shapes of the rings in the SGUP and DSG (see Fig. 7b). For small rings, the $l_2/l_3$ of the SGUP decreased from that of the OSG, while the ratios of the DSG and OSG were almost identical. This difference between SGUP and DSG indicates that cold compression deforms the small rings, whereas hot compression retains the shape of the small rings. By contrast, for large rings, $l_2/l_3$ of the DSG is smaller than that of the SGUP-the rings in the DSG are more deformed than those in the SGUP. The difference in ring deformation between SGUP and DSG was attributed to the presence or absence of Si–O bond recombination. Because Si–O bond recombination is absent in the SGUP, all rings are forcefully deformed with a significant reduction in the Si–O–Si angles (Fig. 3e), and the ring shapes are distorted (Figs. 6d, 7c). By contrast, the Si–O bond recombination in the DSG through hot compression suppressed the considerable reduction in the Si–O–Si angles and the distorted shape of the rings by preferentially deforming the large rings. This is because large rings can adopt various configurations to allow large Si–O–Si angles, as discussed previously. The large structural relaxation caused by hot compression deforms the large rings into elongated shapes and retains the shape of the small rings, as shown in Fig. 7c, preventing a local energy increase resulting from small Si–O–Si angles (see Fig. 4b).

The primary contribution to the formation of the FSDP is thought to originate from the length scale of $l_2$, since the $l_2$ distribution contains primarily the length scale of the FSDP, $Q_1 = 2\pi /\Delta r = 1.5\sim 1.8$, where $\Delta r \simeq 3.5\sim 4.2$ (see Figs. 5, 7d). By examining the $l_2$ distributions of the ring sizes, we elucidated the relationship between ring deformation and the change in MRO in high-density silica glass. The $l_2$ distributions of the SGUP exhibited almost uniform shifts toward a smaller length scale compared to those of the OSG, as shown in Fig. 7e. The differences in the length scales between the different ring sizes of the SGUP were similar to those of the OSG, indicating that the quasi-periodicity of the cage boundaries was basically identical to that of the OSG. This result agrees with the analysis of the partial differential distribution functions and ring center structure factor of the SGUP. By contrast, the distributions of the smaller rings of the DSG than 5-member are almost unchanged compared to those of the OSG, whereas those of the larger rings than 7-member largely shifted to a small length scale from those of the OSG. Thus, the $l_2$ distributions of the rings of the DSG exhibit a large overlap compared to those of the SGUP and OSG, indicating the aligned length scales of rings with different sizes (see also the schematic figure of the DSG in Fig. 7c). The aligned length scales of the rings in the DSG are directly linked to the deformation of the rings in the DSG, i.e., small rings in the DSG tend to maintain their shape, whereas large rings tend to become elongated. Consequently, the length scales ($l_2$) of the different rings are rendered close, and the aligned length scales of the rings enhance the quasi-periodicity of the SiO$_4$ network in the DSG.

## Conclusion

We have performed extensive MLMD simulations to explore the structural origin of MRO in high-density silica glass. Our MLMD simulation reasonably reproduced the structural properties of high-density silica glasses observed in experiments, including the reduction and enhancement of the FSDP depending on the compression temperature. Based on the structures of the simulated high-density silica glasses, we investigated the structural origin of the difference in the FSDPs between SGUP and DSG, which have almost the same density. Two primary factors were identified that influence the reduction or development of the FSDP in these high-density silica glasses. The first relates to the shape of boundary surfaces of cages formed by rings, quantifiable through persistent diagrams. The second factor is associated with the quasi-periodicity in the SiO$_4$ tetrahedral network, which correlates with the length scale of the rings. Under cold compression for the SGUP, there was no recombination of the Si–O bond, meaning that the topology of the SiO$_4$ tetrahedral network remains unchanged. This observation was supported by the partial differential distribution, ring statistics, and the ring center pair distribution function. The reduction in FSDP within the SGUP is attributed to the ring shape distortion caused by compression, which in turn weakens the quasi-Bragg diffraction between the boundary surfaces of successive cages in the SiO$_4$ tetrahedral network. For the DSG created by hot compression, the recombination of the Si–O bond created and deformed large rings. The creation and deformation of the large rings occurred in order to avoid a local energy increase associated with a significant reduction of the Si–O–Si angles by densification. This is because large rings have flexibility, allowing for larger Si–O–Si angles in a high-density state, whereas the deformation of smaller rings inevitably results in a reduction of the Si–O–Si angles. The large rings tended to deform into elongated shapes, characterized by significant changes in their aspect ratios ($l_2/l_3$), while the shape of smaller rings remains almost unchanged. Consequently, the length scales of small to large rings ($l_2$) became close, and quasi-periodicity developed. The large structural relaxation through high-temperature compression caused the cage boundary surfaces to form rings with well-aligned length scales and small distortions, which contributed to the enhancement of the FSDP.

## Methods

### Machine learning molecular dynamics

A machine-learning potential based on the Behler-Parrinello type neural network^45,46^ was created using the n2p2 code. The details and validations of the proposed machine-learning potential are provided in the Supplementary Information. MD simulations with the machine-learning potential were performed using the LAMMPS code^68^ with an n2p2 interface. A Nosè-Hoover thermostat^69,70^ and a Parrinello-Rahman barostat^71,72^ were used to control the temperature and pressure of the systems, respectively.

### Simulated silica glass

Ordinary silica glass (OSG) structures were created via melt quenching simulations. We first conducted an *NPT* simulation of liquid silica with 1728 atoms at 3500 K. The time step and total simulation time were 1 fs and 500 ps, respectively. Five configurations of liquid silica were selected at 100 ps intervals from the MD trajectories. A melt-quenching simulation was then performed for the five structures from temperatures of 3500–300 K at a cooling rate of 0.5 K/ps. In this study, we calculated all the physical quantities of simulated silica glasses from MD trajectories generated via *NPT* simulations with 100 ps run at 300 K. In addition, we assumed the ensemble average using five different configurations of silica glass to improve the statistical certainty of the physical quantities.

We also created two types of simulated high-density silica glass: SGUP and DSG. The SGUPs were prepared by applying various pressures (2.5, 5.0, 7.5, and 10 GPa) to the simulated OSG with a simulation time of 100 ps at 300 K. The DSGs were created by compressing the simulated OSG at high temperatures (1000, 1500, 2000, 2500, 3000, and 3500 K) and pressures (2.5, 5.0, 7.5, and 10 GPa). The OSG was compressed at the target pressure and temperature for 100 ps, and then cooled to 300 K at a rate of 0.5 K/ps. After the quenching simulation, the applied pressure was gradually released to 0 GPa for 100 ps. The physical properties of the simulated high-density silica glasses were computed in the same manner as those for the OSG.

### Faber–Ziman total structure factor

We calculated the Faber–Ziman total structure factor from the partial pair distribution function $g_{\alpha \beta }(r)$ of the simulated silica glass as

4 $$\begin{aligned}{} & {} S_{\textrm FZ}(Q) = \sum _{\alpha ,\beta }\frac{c_{\alpha }c_{\beta }b_{\alpha }(Q)b_{\beta }(Q)}{\langle b(Q) \rangle ^2}S_{\alpha \beta }(Q) , \end{aligned}$$

5 $$\begin{aligned}{} & {} S_{\alpha \beta }(Q) = 1+4\pi \rho _{0}\int dr r^{2}\frac{\sin (Qr)}{Qr}\left( g_{\alpha \beta }(r)-1 \right) , \end{aligned}$$

where $\rho _{0}$ is the density of the silica glass, $c_{\alpha }$ is the concentration of chemical species ($\alpha =\text {Si,O}$), and $b_{\alpha }(Q)$ is the neutron (or X-ray) scattering factor for the chemical species, and $\langle b(Q) \rangle$ denotes the average of the scattering factor as $\langle b(Q) \rangle \equiv \sum _{\alpha }c_{\alpha }b_{\alpha }(Q)$ . We used neutron scattering lengths, $b_{\textrm Si}=4.1491$ and $b_{\textrm O}=5.803$, to calculate the total structure factor of the ND. The *Q*-dependent scattering factors $b_{\alpha =\text {Si,O}}(Q)$ for the calculation of the total structure factor of the XRD were taken from the reference^73^.

### Topological analysis

The topology embedded in the SiO$_4$ network in high-density silica glass was analyzed using ring statistics and persistence homology analyses^21,74^. The ring size distribution was computed using shortest path analysis^75^. We defined the edges of an undirected graph from the Si–O bonds and collected the shortest rings connected to an Si atom using a depth-first search. Persistence homology analysis was performed using the HomCloud package^74^. The mathematical details of persistence homology can be found in the references^21,74^. In this method, we generate an atomic ball with radius $r_{i}(\alpha ) = \sqrt{\alpha +r_{i}^2}$, where $r_{i}$ is the initial radius of each atom and the parameter $\alpha$ is an adjustable parameter. By varying $\alpha$ from zero to a sufficiently large value, we can detect the loops embedded in the atomic configuration at each $\alpha$. The detected loops are characterized by birth and death scales (*b* and *d*), at which the loop first appears ($\alpha ^2 = b$) and disappears ($\alpha ^2 = d$). Subsequently, the persistence diagram is given as a 2D histogram counting the number of loops on the birth-death plane, which provides quantitative information on both the shape and length of the loops embedded in the SiO$_{4}$ network. In this study, the input radii of oxygen and silicon atoms ($r_{\textrm O}$ and $r_{\textrm Si}$) were determined from the O–O and Si–O bond lengths ($d_{\textrm OO}$ and $d_{\textrm SiO}$) as $r_{\textrm O} = d_{\textrm OO}/2$ and $r_{\textrm Si}=d_{\textrm SiO}-r_{\textrm O}$^21^.

### Supplementary Information

Supplementary Information.

## Data Availability

The machine learning potentials and the datasets generated during the current study are available from the corresponding author on reasonable request.
