# Supplementary material for: Machine learning molecular dynamics reveals the structural origin of the first sharp diffraction peak in high-density silica glasses
Source: Sci Rep. 2023 Nov 16;13:18721. doi: 10.1038/s41598-023-44732-0 (PMC10654503; doi:10.1038/s41598-023-44732-0)
Supplement: Supplementary file 1 — Supplementary Information. [file 41598_2023_44732_MOESM1_ESM.pdf]

Supplementary Information for "Machine Learning Molecular Dynamics Reveals the Structural Origin of the First Sharp Diffraction Peak in High-Density Silica Glasses"

Keita Kobayashi[a] Masahiko Okumura[a], Hiroki Nakamura[a], Mitsuhiro Itakura[a], Masahiko Machida[a], Shingo Urata[b], and Kentaro Suzuya[c]

1 Machine learning potential of silica

In this appendix, we show the details of the present machine-learning potential (MLP) and the validation of MLP for crystal structures of silica.

1.1 Details of the MLP in the present work

The reference data of MLP of silica was generated by DFT calculation using Vienna *Ab initio* Simulation Package (VASP). The quartz, cristobalite, tridymite, stishovite, amorphous, and liquid structures were used for constructing the DFT reference data. First, we generated various configurations for the above structures using MD simulations with Tersoff potential[1]. The energies and forces for the configurations were then reevaluated by DFT calculation and used as the DFT reference data. We employed the strongly constrained and appropriately normed meta-GGA exchange-correlation functional for the DFT calculation with an energy cutoff of 500 eV and k-spacing 0.25 Å^{-1}. We also conducted DFT-NPT simulations for the quartz, cristobalite, tridymite, stishovite, and liquid structures at 300, 500, 1000, 3000, and 5000 K. The DFT-NPT simulations for the crystal structures at 5000 K were performed until the solids melted completely. We picked up the structures from the MD trajectories at 25 fs intervals, and the total number of the DFT reference data for making the MLP was 21338 structures. 90% of the DFT reference data was assigned to training data and the remaining 10% to test data, respectively.

We used the n2p2 code[2,3] for training MLP based on the Behler-Parrinello type neural network[4,5]. We adopted the following symmetry functions as the descriptors of the distances

[a] Center for Computational Science and e-Systems, Japan Atomic Energy Agency, Kashiwa, Chiba 277–0871, Japan
[b] Innovative Technology Research Center, AGC Inc., 1150 Hazawa-cho, Kanagawa-ku Yokohama, Kanagawa 221-8755, Japan
[c] Materials & Life Science Division, J-PARC Center, Japan Atomic Energy Agency, Tokai, Ibaraki 319-1195, Japan

and the angles of atoms, respectively, i.e.,

$$G_i^{(\mathrm{R})} = \sum_j e^{-\eta^{(R)}(R_{ij}-R_\mathrm{s})^2} f_\mathrm{c}(R_{ij})\,, \tag{1}$$

$$G_i^{(\mathrm{A})} = 2^{1-\xi} \sum_{j\neq i} \sum_{k\neq i,j} (1+\lambda\cos\theta_{ijk})^\xi \, e^{-\eta^{(A)}(R_{ij}^2+R_{ik}^2+R_{jk}^2)} \times f_\mathrm{c}(R_{ij}) f_\mathrm{c}(R_{ik}) f_\mathrm{c}(R_{jk})\,, \tag{2}$$

with the cutoff function[2]

$$f_\mathrm{c}(R) = \begin{cases} R^3(R(15-6R)-10) & R \leq R_\mathrm{c} \\ 0 & \text{for} \quad R_\mathrm{c} < R \end{cases} \,, \tag{3}$$

where R_{ij} is the distance between the i-th and j-th atoms, and θ_{ijk} is the angle between i-j and i-k atom bonds . The cutoff radius R_c for $G_i^{(\mathrm{R})}$ and $G_i^{(\mathrm{A})}$ were taken as 8.0 Å and 6.5 Å, respectively. The other parameters of symmetry functions ($\eta^{(R)}$, $\eta^{(A)}$, R_s, λ, and ξ) were selected by CUR decomposition[6]. We used two hidden layers with softplus activation functions with 20 nodes. The root mean square errors (RMSE) of energy and force for the training and test data were summarized in table S1.

Table S1: RMSE of MLP for the training and test data.

	Training (90%)	Test (10%)
Energy (meV/atom)	1.536	1.475
Force (10^{-2} eV/Å)	9.930	9.306

1.2 Validation of MLP for crystal structures of silica

Table S2: The lattice constants of various crystal structures of silica obtained by DFT, MLP, and experiment (EXP). The round bracket $(\cdot)_{\mathrm{EXP(DFT)}}$ represents the percentage error for EXP and DFT, respectively.

		a [Å]	b [Å]	c [Å]
	DFT	4.914	4.914	5.419
		$(0.04)_{\mathrm{EXP}}$	$(0.04)_{\mathrm{Exp}}$	$(0.25)_{\mathrm{EXP}}$
α-quartz	MLP	4.900	4.911	5.394
		$(0.28)_{\mathrm{DFT}}$	$(0.07)_{\mathrm{DFT}}$	$(0.46)_{\mathrm{DFT}}$
	Exp [7]	4.916	4.916	5.405
	DFT	4.974	4.974	6.921
		$(0.06)_{\mathrm{EXP}}$	$(0.06)_{\mathrm{EXP}}$	$(0.02)_{\mathrm{EXP}}$
α-cristoblite	MLP	5.046	5.046	7.038
		$(1.43)_{\mathrm{DFT}}$	$(1.43)_{\mathrm{DFT}}$	$(1.43)_{\mathrm{DFT}}$
	Exp [8]	4.972	4.972	6.922
	DFT	8.824	4.939	8.219
		$(0.96)_{\mathrm{EXP}}$	$(2.05)_{\mathrm{EXP}}$	$(0.26)_{\mathrm{EXP}}$
α-tridymite	MLP	8.831	4.941	8.215
		$(0.08)_{\mathrm{DFT}}$	$(0.03)_{\mathrm{DFT}}$	$(0.96)_{\mathrm{DFT}}$
	Exp [9]	8.740	5.040	8.240
	DFT	4.199	4.199	2.689
		$(0.18)_{\mathrm{EXP}}$	$(0.12)_{\mathrm{EXP}}$	$(0.30)_{\mathrm{EXP}}$
stishovite	MLP	4.155	4.155	2.663
		$(1.05)_{\mathrm{DFT}}$	$(1.05)_{\mathrm{DFT}}$	$(0.04)_{\mathrm{DFT}}$
	Exp [10]	4.179	4.179	2.666
	DFT	7.144	12.401	7.197
		$(0.54)_{\mathrm{EXP}}$	$(0.31)_{\mathrm{EXP}}$	$(0.26)_{\mathrm{EXP}}$
coesite	MLP	7.182	12.362	7.178
		$(0.53)_{\mathrm{DFT}}$	$(0.32)_{\mathrm{DFT}}$	$(0.26)_{\mathrm{DFT}}$
	Exp [11]	7.136	12.384	7.186
	DFT	4.090	5.052	4.508
		$(0.18)_{\mathrm{EXP}}$	$(0.12)_{\mathrm{EXP}}$	$(0.30)_{\mathrm{EXP}}$
seifertite	MLP	4.061	5.003	4.476
		$(0.69)_{\mathrm{DFT}}$	$(0.97)_{\mathrm{DFT}}$	$(0.71)_{\mathrm{DFT}}$
	Exp [12]	4.097	5.046	4.495

In the main text, we have shown that the present MLP of silica well reproduces the structural properties of silica glass. Here, we show the performance of the MLP for the crystal phase of silica. Table S2 shows the lattice constants of various crystal phases obtained by structural optimization using DFT and MLP. The lattice constants computed by DFT based on SCAN are in quite good agreement with the experimental data. The present MLP is able to reproduce the DFT results very well, with the maximum error for DFT data being only 1.43 for α-cristobalite.

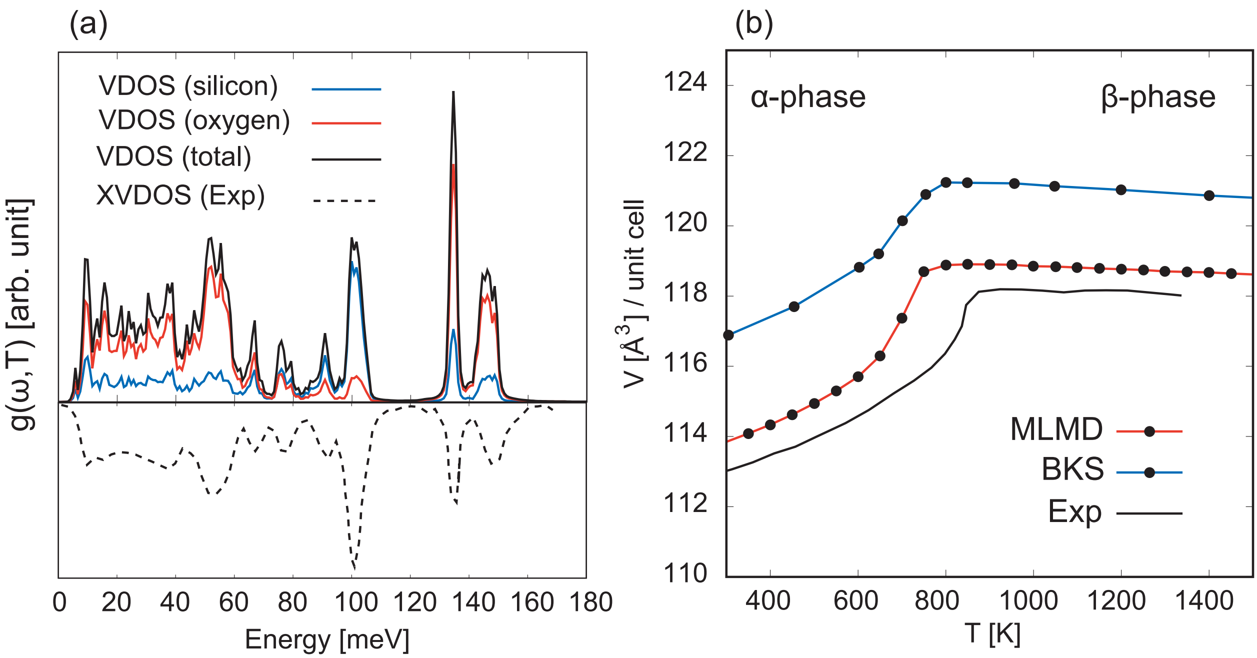

Fig. S1: (a) VDOS of α-quartz at 300 K obtained by MLMD. Blue, red, and black solid lines show the silicon, oxygen, and total VDOS, respectively. Black dashed line denotes the experimental data of inelastic X-ray scattering [13]. (b) Temperature dependence of the volume of quartz obtained by MLMD, MD with BKS potential [14], and experiment (Exp) [15].

For the validation of our present MLP, we also compute the finite temperature properties of quartz with 1125 atoms. Firstly, we evaluate vibrational density of state (VDOS) at finite temperature using the Fourier transform of the velocity autocorrelation function as

$$g(\omega, T) = \frac{1}{N k_{\mathrm{B}} T} \sum_{j=1}^{N} m_j \int_{-\infty}^{\infty} dt \langle \boldsymbol{v}_j(t) \cdot \boldsymbol{v}_j(0) \rangle e^{i\omega t} \,, \tag{4}$$

where ω, T, N, k_{B}, m_j, and $\boldsymbol{v}_j$ are the frequency, temperature, the number of atoms, the Boltzmann constant, the mass of the j-th atom, and the velocity of the j-th atom, respectively. To obtain the velocities of atoms at each time step from MD trajectory, we conducted MLMD-NVE simulation at 300 K with 0.5 fs time step and 200 ps simulation period. The peak positions of the VDOS obtained by MLMD agree well with the experimental data [13] as shown in Fig.S1(a). Next, we investigate the phase transition from α- to β-quartz using MLMD-NPT simulation. Fig.S1(b) shows temperature dependence of the volume of quartz per unit cell. The quartz in α-phase shows positive thermal expansion, whereas that in β-phase reveals negative thermal expansion. Although the curve of volume change obtained by MLMD slightly shift to a lower temperature than the experimental curve, the values are quite close to experimental data. From the above validations, we confirmed that the present MLP enables us to conduct accurate calculation not only for the amorphous phase but also for the crystal phase of silica.

2 Additional data of simulated high-density silica glass

2.1 Comparison of structure factors of high-density silica glass obtained by MLMD simulation and experiments

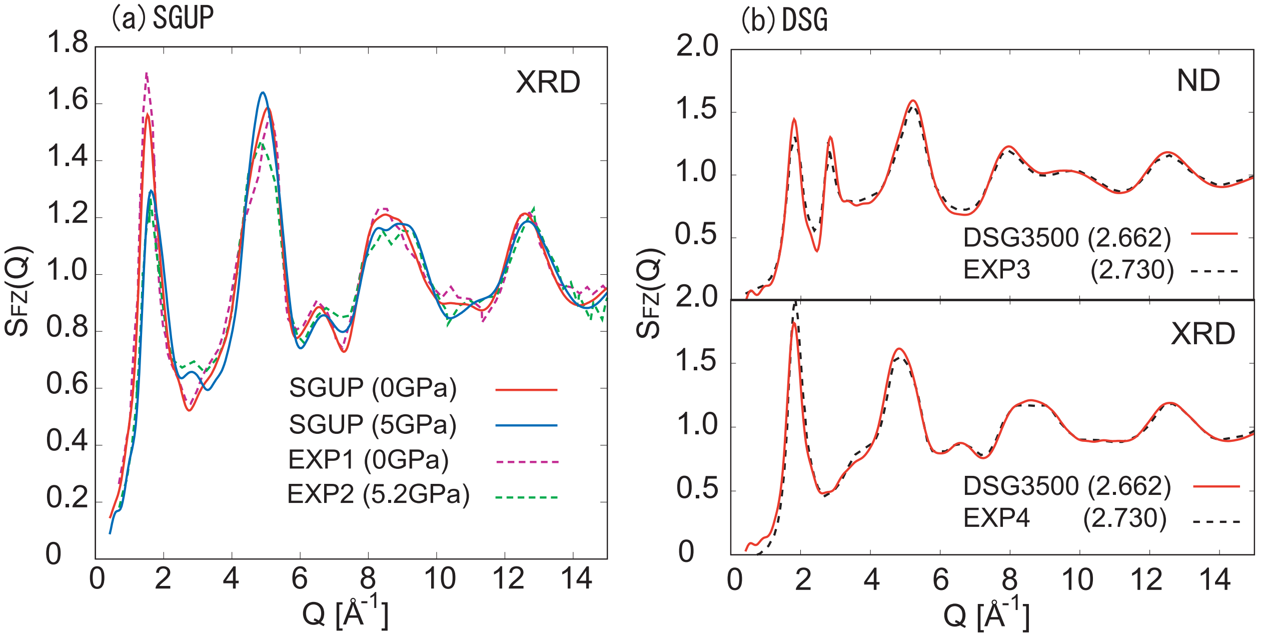

Fig. S2: (a) Faber–Ziman total structure factors of XRD of the SGUP. Solid lines (SGUP) are the results obtained by MLMD simulation and the dashed lines (EXP1 and EXP2) are experimental data taken from supplementary information of reference [16]. (b) Faber–Ziman total structure factors of ND and XRD of the DSG. Solid lines (DSG3500) is the result computed by MLMD simulation and the dashed lines (EXP3 and EXP4) are experimental results [17]. The values in (·) in the legends of (b) are the density of silica glass [g/cm^3].

Fig.S2(a) shows the Faber–Ziman total structure factors of the SGUP obtained by the MLMD and high-pressure XRD experiment [16]. Although some discrepancies exist with regard to the height of peaks, the MLMD simulation is able to reasonably reproduces the high-pressure XRD experimental data, including a small peak around 2.9 Å, which can not be reproduced by some classical force field [18,19] (refer to supplementary information of reference [16]) Fig.S2(b) also shows the total structure factors of the DSG obtained by the MLMD, and experiments [17]. Here, we compared experimental data with calculated data of the DSG3500, which has a density close to the experimental one. The results of the MLMD simulation agree well with the experimental data of the DSG created by hot compression.

2.2 Partial structure factor of silica glasses

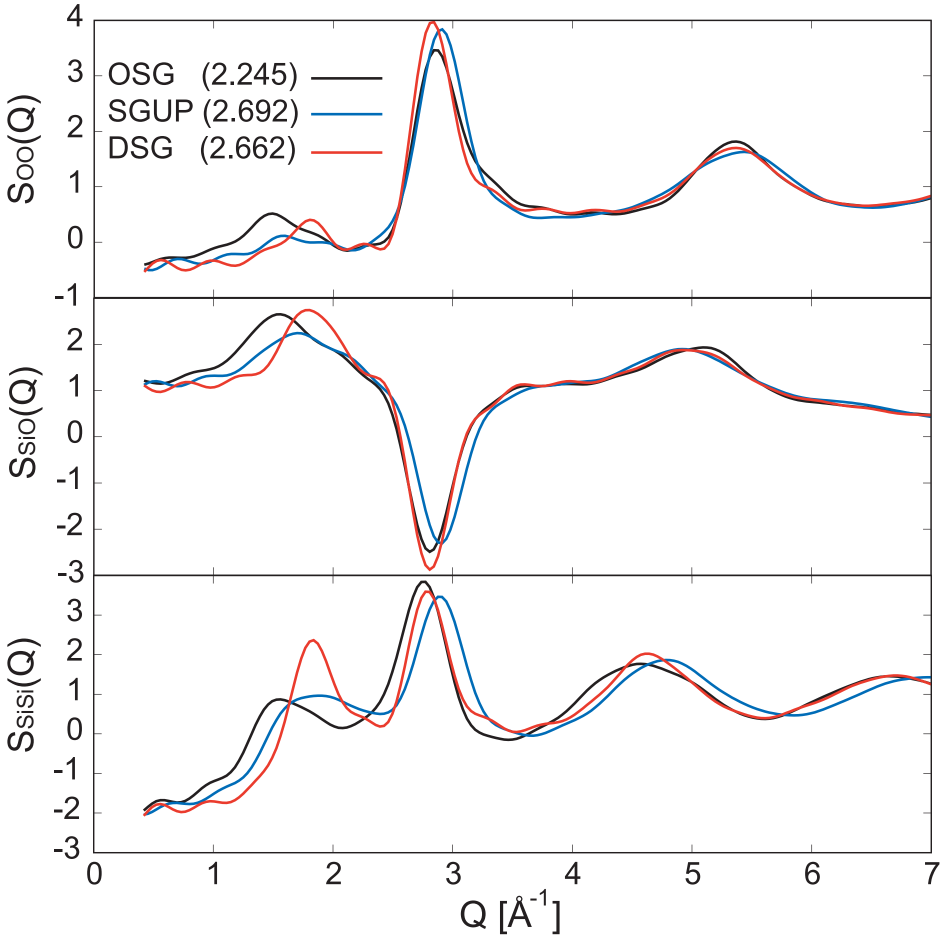

Fig. S3: Partial structure factors for the O-O, Si-O, and Si-Si pair. The black, blue, and red lines are the structure factors for the OSG, the SGUP, and the DSG, respectively. The values in (·) in the legends are the density of silica glass [g/cm^3].

Fig.S3 shows the partial structure factors $S_{\alpha\beta}(Q)$ of the OSG, SGUP, and DSG. Overall, the first peaks of the partial structure factors in the SGUP decrease and broaden with a peak shift towards a higher scatter vector. In the case of the DSG, the first peak in the Si-Si partial structure factor significantly developed, which primarily contributes to the enhancement of the FSDP in the total structure factor. Although both PPs in the $S_{\rm FZ}(Q)$ for the SGUP and DSG become sharper than that of the OSG (see the upper panel of Fig5.(a)), the contribution of each $S_{\alpha\beta}(Q)$ to the $S_{\rm FZ}(Q)$ differs in the SGUP and DSG. As density increases, the second negative peak intensity in the $S_{\rm SiO}(Q)$ behaves oppositely: the second negative peak of the SGUP increases, while that of the DSG decreases. The small peak of the XRD $S_{\rm FZ}(Q)$ around 2.9 Å for the SGUP, which is absent in the DSG (see Fig.S2), can be ascribed to the increase of the second negative peak in the $S_{\rm SiO}(Q)$.

2.3 Ring center partial differential distribution function and structure factor for the SGUPs and the DSGs with various density

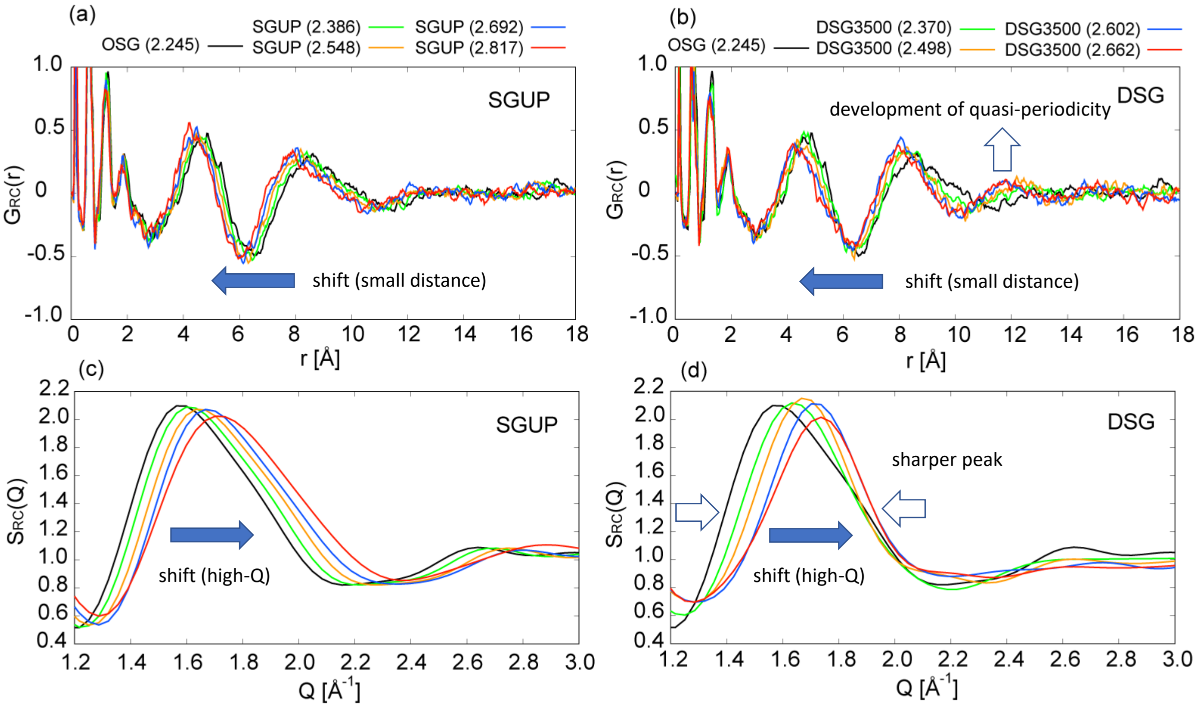

Fig. S4: (a) and (b) Ring center differential distribution function $G_{\mathrm{RC}}(r)$ for OSG, SGUP, and DSG3500. (c) and (d) Ring center structure factor $S_{\mathrm{RC}}(Q)$ for OSG, SGUP, and DSG3500. The values in $(\cdot)$ in the legends are the density of silica glass [g/cm^3].

Figure S4 shows systematic changes in the ring center differential distribution functions $G_{\mathrm{RC}}(r)$ and structure factor $S_{\mathrm{RC}}(Q)$ of the SGUP and DSG3500. With increasing density, the $G_{\mathrm{RC}}(r)$ peaks of the SGUP in the intermediate range move towards shorter distance, and the peaks of $S_{\mathrm{RC}}(Q)$ shift to a higher scattering vector Q. The shapes of the $S_{\mathrm{RC}}(Q)$ are almost identical to that of the OSG, indicating that the origin of the MRO in the SGUP is the same as that in the OSG, as discussed in the main text. In contrast, the DSGs show a new peak at around 12 Å, in addition to the shifts of the medium-distance peaks in $G_{\mathrm{RC}}(r)$. Furthermore, the $S_{\mathrm{RC}}(Q)$ peaks become sharper as shown in Fig.S4(d), indicating the development of quasi-periodicity in the DSGs.

2.4 Ring center structure factor and persistence diagram for the DSGs with almost the same density

We present an analysis for the FSPDs in the DSGs with similar densities. Fig.S5 shows the ring center structure factors $S_{\mathrm{RC}}(Q)$ and the probability distributions of the death scale $P(d)$ on the L_1 band in the persistence diagram. The peak of the $S_{\mathrm{RC}}(Q)$ becomes sharper as compression temperature increases, and the peak positions are located around $Q \simeq 1.70$,

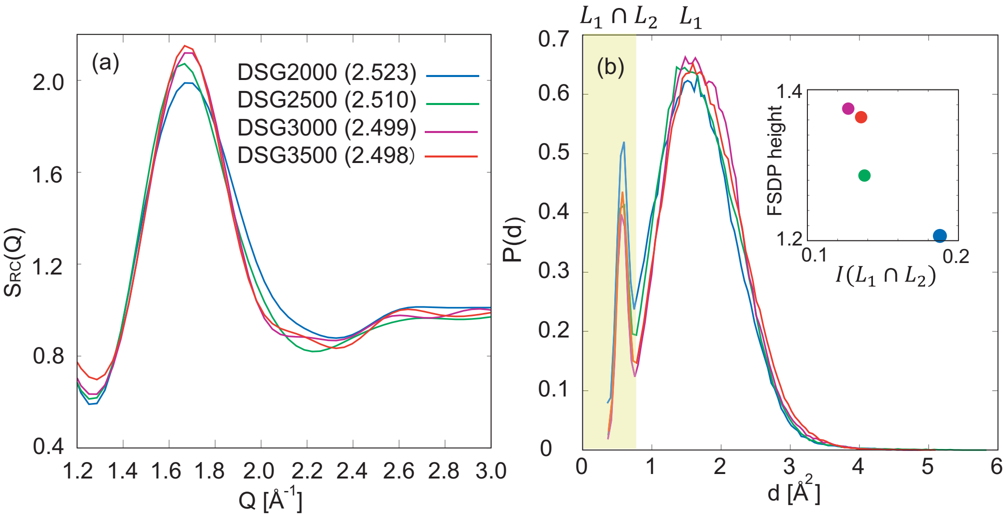

Fig. S5: (a) Ring center structure factor $S_{\mathrm{RC}}(Q)$ for DSGs compressed at 2000, 2500, 3000, and 3500 K (DSG2000, DSG2500, DSG3000, and DSG3500), respectively. (b) Probablility distribution of death scale $P(d)$ on the L_1 band of the persistence diagram for DSG2000, DSG2500, DSG3000, and DSG3500. The inset of (c) shows the scatter plot the height of the FSDP and the intensity of the peak on the $L_1 \cap L_2$ band, $I(L_1 \cap L_2)$, which is defined as the integration of $P(d)$ from $d = 0$ to the first minimum of the $P(d)$. The blue, green, purple, and red lines (points) denote the results of DSG2000, DSG2500, DSG3000, and DSG3500, respectively. The values in $(\cdot)$ in the legends are the density of silica glass [g/cm^3].

which roughly coincide with those of $S_{\mathrm{FZ}}(Q)$ in Fig.5(b). The peak sharpening indicates the development of the quasi-periodicity between the cage boundaries by the recombination of the SiO_4 network under high-temperature compression. Focusing on the shape of the rings, the large differences between the DSGs are observed in the peak intensities on the $L_1 \cap L_2$ band (the yellow region in Fig.S5(b)), indicating that the ratio of distorted rings in the DSGs changes by the compression temperature. Since the ratio of distorted rings in the DSGs is considered to affect the FSDP intensity as discussed in the main text, we investigate the relationship between the intensity of the peak on the $L_1 \cap L_2$ bands and the FSDP height. The inset of Fig.S5(b) shows the scatter plot of the intensity of the $L_1 \cap L_2$ band peak and the FSDPs height, revealing a correlation between the two. The DSGs compressed at higher temperatures are accompanied with large structural relaxation, which make more aligned cage boundary surfaces formed by rings with small distortion contributing to the enhancement of the peak intensity of the FSDP.

2.5 Additional information of the ring approximated as a cuboid

In the main text, we have presented the change in the aspect ratio l_2/l_3, which characterizes the deformation of rings within a ring pseudo-plane. In this supplementary material, we show the change of the ratio of thickness l_1 and average length $l_\perp = (l_2 + l_3)/2$ of the ring pseudo-plane for different ring sizes in Fig.6(a). The ratio $l_1/l_\perp$ approaches zero when the ring pseudo-plane is almost flat. The difference in $l_1/l_\perp$ of the DSG for small rings compared to that of the OSG is smaller than that of the SGUP. Conversely, the difference in $l_1/l_\perp$ of

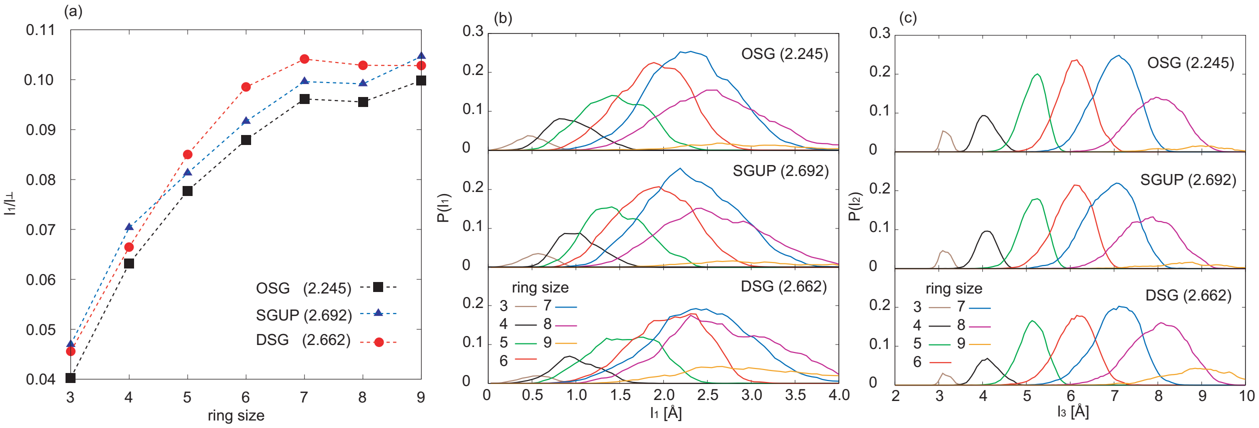

Fig. S6: (a) Averaged ratio of $l_1/l_\perp$ for each ring size. (b) and (c) show the distribution of l_1 and l_3 calculated for each ring size, respectively. The values in $(\cdot)$ in the legends are the density of silica glass [g/cm^3].

the DSG for larger rings tends to be larger than that of the SGUP. The ring size-dependent changes in $l_1/l_\perp$ of the DSG are the same as those of l_2/l_3, meaning that larger rings in the DSG are more likely to deform to avoid a substantial reduction in the Si-O-Si angle, as explained in the main text. Additionally, the distribution of l_1 and l_3 calculated for each ring size is shown in Fig.6(b) and (c). The ring size-dependent of the $l_1/l_\perp$ of the DSG is the same as those of l_2/l_3, , meaning that larger rings in the DSG are more likely to deform to avoid a substantial reduction in the Si-O-Si angle, as explained in the main text.

We also show the distribution of l_1 and l_3 calculated for each ring size in Fig.6(b) and (c) as additional information.

References

[1] J. Tersoff. New empirical approach for the structure and energy of covalent systems. *Phys. Rev. B*, Vol. 37, pp. 6991–7000, Apr 1988.

[2] Andreas Singraber, Jörg Behler, and Christoph Dellago. Library-based lammps implementation of high-dimensional neural network potentials. *Journal of Chemical Theory and Computation*, Vol. 15, No. 3, pp. 1827–1840, 2019.

[3] Andreas Singraber, Tobias Morawietz, Jörg Behler, and Christoph Dellago. Parallel multistream training of high-dimensional neural network potentials. *Journal of Chemical Theory and Computation*, Vol. 15, No. 5, pp. 3075–3092, 2019.

[4] Jörg Behler and Michele Parrinello. Generalized neural-network representation of high-dimensional potential-energy surfaces. *Phys. Rev. Lett.*, Vol. 98, p. 146401, Apr 2007.

[5] Jörg Behler. Constructing high-dimensional neural network potentials: A tutorial review. *International Journal of Quantum Chemistry*, Vol. 115, No. 16, pp. 1032–1050, 2015.

[6] Giulio Imbalzano, Andrea Anelli, Daniele Giofré, Sinja Klees, Jörg Behler, and Michele Ceriotti. Automatic selection of atomic fingerprints and reference configurations for machine-learning potentials. *The Journal of Chemical Physics*, Vol. 148, No. 24, p. 241730, 2018.

[7] Louise Levien, Charles T. Prewitt, and Donald J. Weidner. Structure and elastic properties of quartz at pressure. *American Mineralogist*, Vol. 65, No. 9-10, pp. 920–930, 10 1980.

[8] R. T. Downs and D. C. Palmer. The pressure behavior of α cristobalite. *American Mineralogist*, Vol. 79, No. 1-2, pp. 9–14, 02 1994.

[9] W. A. Dollase. The crystal structure at 220°C of orthorhombic high tridymite from the Steinbach meteorite. *Acta Crystallographica*, Vol. 23, No. 4, pp. 617–623, 1967.

[10] Nancy L. Ross, Jinfu Shu, and Robert M. Hazen. High-pressure crystal chemistry of stishovite. *American Mineralogist*, Vol. 75, No. 7-8, pp. 739–747, 08 1990.

[11] Joseph R. Smyth, Joseph V. Smith, Gilberto. Artioli, and Ake. Kvick. Crystal structure of coesite, a high-pressure form of silica, at 15 and 298 K from single-crystal neutron and x-ray diffraction data: test of bonding models. *The Journal of Physical Chemistry*, Vol. 91, No. 4, pp. 988–992, 1987.

[12] Przemyslaw Dera, Charles T. Prewitt, Nabil Z. Boctor, and Russell J. Hemley. Characterization of a high-pressure phase of silica from the martian meteorite Shergotty. *American Mineralogist*, Vol. 87, No. 7, pp. 1018–1023, 2002.

[13] Alexei Bosak, Michael Krisch, Dmitry Chernyshov, Björn Winkler, Victor Milman, Keith Refson, and Clemens Schulze-Briese. New insights into the lattice dynamics of α-quartz. *Zeitschrift für Kristallographie - Crystalline Materials*, Vol. 227, No. 2, pp. 84–91, 2012.

[14] Daniel Herzbach, Kurt Binder, and Martin H. Müser. Comparison of model potentials for molecular-dynamics simulations of silica. *The Journal of Chemical Physics*, Vol. 123, No. 12, p. 124711, 2005.

[15] Michael A. Carpenter, Ekhard K.H. Salje, Ann Graeme-Barber, Martin T. Dove, and Kevin S. Knight. Calibration of excess thermodynamic properties and elastic constant variations associated with the $\alpha \leftrightarrow \beta$ phase transition in quartz. *American Mineralogist*, Vol. 83, No. 1, pp. 2–22, 1998.

[16] Yoshio Kono, Koji Ohara, Nozomi Kondo, Hiroki Yamada, Satoshi Hiroi, Fumiya Noritake, Kiyofumi Nitta, Oki Sekizawa, Yuji Higo, Yoshinori Tange, Hirokatsu Yumoto, Takahisa Koyama, Hiroshi Yamazaki, Yasunori Senba, Haruhiko Ohashi, Shunji Goto, Ichiro Inoue, Yujiro Hayashi, Kenji Tamasaku, and Makina Yabashi. Experimental evidence of tetrahedral symmetry breaking in SiO_2 glass under pressure. *Nature Communications*, Vol. 13, , 04 2022.

[17] Yohei Onodera, Shinji Kohara, Philip S. Salmon, Akihiko Hirata, Norimasa Nishiyama, Suguru Kitani, Anita Zeidler, Motoki Shiga, Atsunobu Masuno, Hiroyuki Inoue, Shuta Tahara, Annalisa Polidori, Henry E. Fischer, Tatsuya Mori, Seiji Kojima, Hitoshi Kawaji, Alexander I. Kolesnikov, Matthew B. Stone, Matthew G. Tucker, Marshall T. McDonnell, Alex C. Hannon, Yasuaki Hiraoka, Ippei Obayashi, Takenobu Nakamura, Jaakko Akola, Yasuhiro Fujii, Koji Ohara, Takashi Taniguchi, and Osami Sakata. Structure and properties of densified silica glass: characterizing the order within disorder. *NPG Asia Materials*, Vol. 12, No. 1, p. 85, Dec 2020.

[18] B. W. H. van Beest, G. J. Kramer, and R. A. van Santen. Force fields for silicas and aluminophosphates based on ab initio calculations. *Phys. Rev. Lett.*, Vol. 64, pp. 1955–1958, Apr 1990.

[19] Thiruvilla S Mahadevan, Wei Sun, and Jincheng Du. Development of water reactive potentials for sodium silicate glasses. *The Journal of Physical Chemistry B*, Vol. 123, No. 20, pp. 4452–4461, 2019.
